# Supplementary material for: Evolution of a fuzzy ribonucleoprotein complex in viral assembly
Source: bioRxiv. 2025 Nov 6:2025.04.26.650775. Originally published 2025 Apr 28. Preprint. [Version 3] doi: 10.1101/2025.04.26.650775 (PMC12190348; doi:10.1101/2025.04.26.650775)

Supplementary Figure S7: Non-reducing SDS-PAGE of reduced and oxidized N:G215C* and N$_\lambda$*. Lanes: 1) oxidized N$_\lambda$*; 2) reduced N$_\lambda$; 3) oxidized N:G215C*; 4) reduced N:G215C.

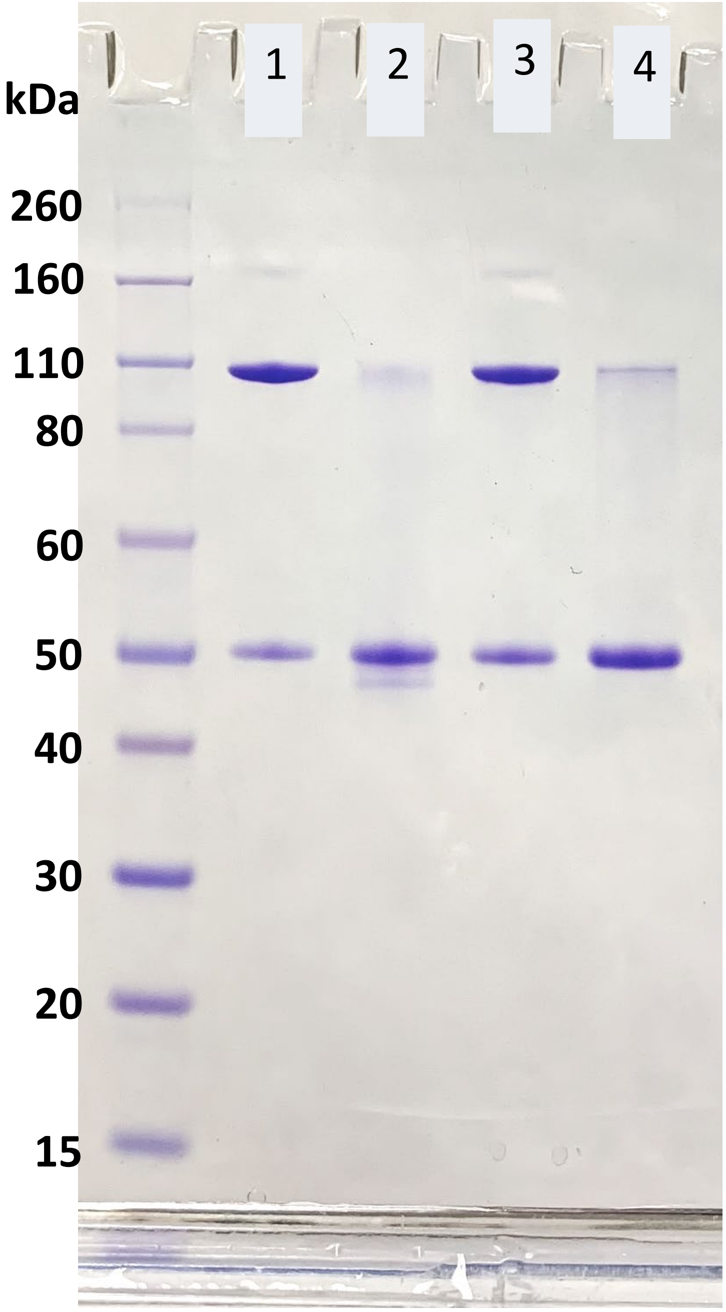

Supplement: Supplement 2 [file media-2.pdf]
