# Supplementary material for: Evolution of a fuzzy ribonucleoprotein complex in viral assembly
Source: bioRxiv. 2025 Nov 6:2025.04.26.650775. Originally published 2025 Apr 28. Preprint. [Version 3] doi: 10.1101/2025.04.26.650775 (PMC12190348; doi:10.1101/2025.04.26.650775)

Supplementary Figure S10: Mutations of N:G215 across the phylogenetic tree of SARS-CoV-2. Shown are all-time global sequence samples with clade labels and color-coded amino acid at position 215, with the ancestral G215 in green and G215C in yellow. The phylogenetic tree was generated by Nextstrain (Hadfield et al., 2018).

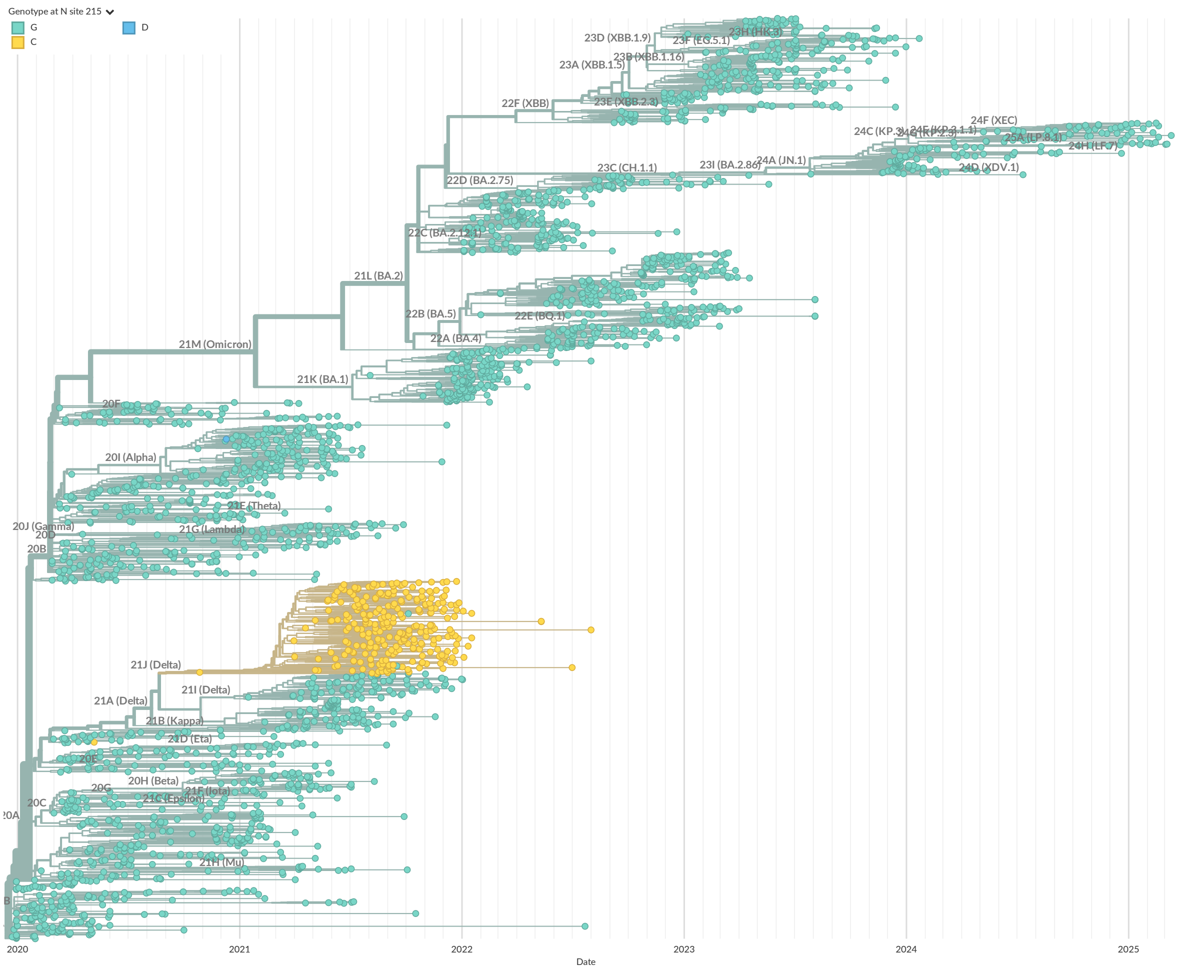

Supplement: Supplement 5 [file media-5.pdf]
