# Supplementary material for: Evolution of a fuzzy ribonucleoprotein complex in viral assembly
Source: bioRxiv. 2025 Nov 6:2025.04.26.650775. Originally published 2025 Apr 28. Preprint. [Version 3] doi: 10.1101/2025.04.26.650775 (PMC12190348; doi:10.1101/2025.04.26.650775)

Supplementary Figure S12: Mutations of N:R203 and N:G204 across the phylogenetic tree of SARS-CoV-2. Shown are global sequence samples mostly representing sequences of the recent 6 month, with clade labels and color-coded amino acid at position 203 and 204. The ancestral combination of R203/G204 is shown in green, the mutation 203M of the Delta VOC in blue, the combination 203K/204R common to Alpha and Omicron VOCs in yellow, and the combination 203K/204P defining in the Omicron XEC variant in orange. The phylogenetic tree was generated by Nextstrain (Hadfield et al., 2018).

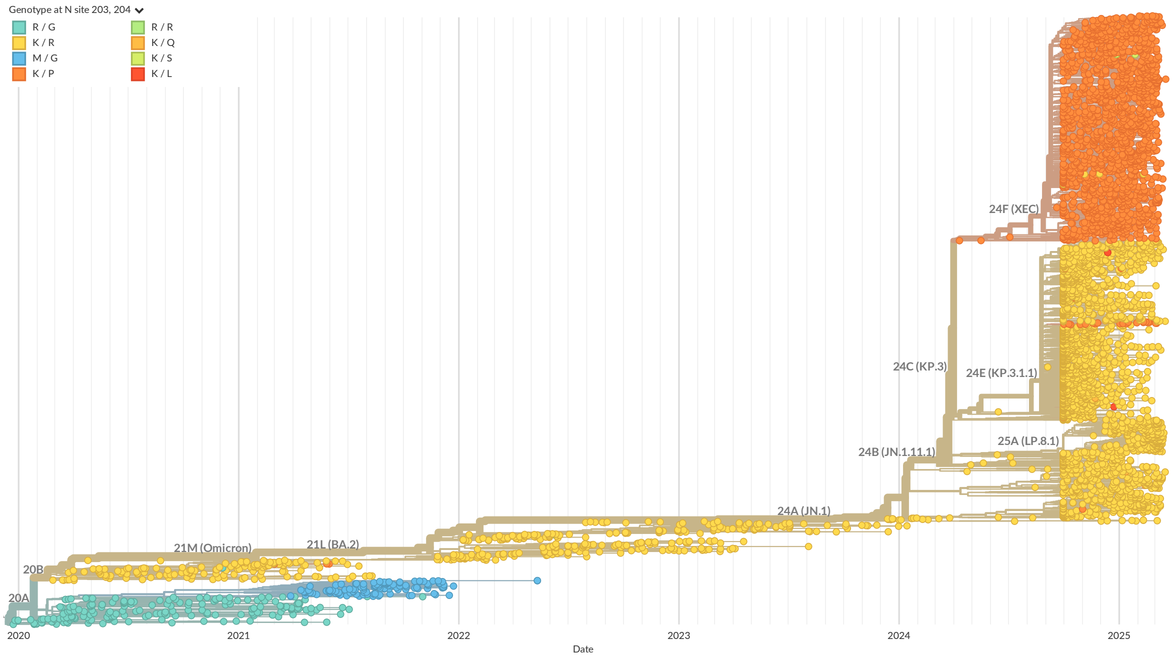

Supplement: Supplement 7 [file media-7.pdf]
