# Supplementary material for: Evolution of a fuzzy ribonucleoprotein complex in viral assembly
Source: bioRxiv. 2025 Nov 6:2025.04.26.650775. Originally published 2025 Apr 28. Preprint. [Version 3] doi: 10.1101/2025.04.26.650775 (PMC12190348; doi:10.1101/2025.04.26.650775)
Supplement: Supplement 8 [file media-8.pdf]

Supplementary Table

Table S1. Peptide and Oligonucleotide Sequences

designation	sequence
N_{1-43} (N-arm)	MSDNGPQNQRNAPRITFGGPSDSTGSNQNGERSGARSKQRRPQ
N_{1-43}:P13L	MSDNGPQNQRNALRITFGGPSDSTGSNQNGERSGARSKQRRPQ
N_{1-43}:P13L,Δ31-33	MSDNGPQNQRNALRITFGGPSDSTGSNQNG---GARSKQRRPQ
$N_{210-246}$ (LRS)	MAGNGGDAALALLLLDRLNQLESKMSGKGQQQQGQTV
$N_{210-246}$:G214C	MAGNCGDAALALLLLDRLNQLESKMSGKGQQQQGQTV
$N_{210-246}$:G215C	MAGNGCDAALALLLLDRLNQLESKMSGKGQQQQGQTV
T_{10}	TTTTTTTTTT
SL7	ACGUGGCUUUGGAGACUCCGUGGAGGAGGUCUUAUCAGAGGCACGU
