# Supplementary material for: Evolution of a fuzzy ribonucleoprotein complex in viral assembly
Source: bioRxiv. 2025 Nov 6:2025.04.26.650775. Originally published 2025 Apr 28. Preprint. [Version 3] doi: 10.1101/2025.04.26.650775 (PMC12190348; doi:10.1101/2025.04.26.650775)

Supplementary Figure S1: Possible heterogeneity of RNPs from alternate configurations of N dimer subunits. Only the N protein scaffold is shown for clarity. The nucleic acid binding domain at N-terminus (NTD) is indicated in blue, the dimerization domain (CTD) in green, and the L-rich region (206-247, LRS) folded into helices is shown as yellow rods. The other disordered regions are shown as black lines. (A) Based on the cartoon of **Figure 1C**, an RNPs is sketched where two of the 6 dimer subunits have flipped LRS orientations, resulting in the swapped position of NTD and CTD subunits. (B) Arrangement of 5 dimer subunits. (C) Arrangement of 7 dimer subunits. (D) $N_{210\text{-}419}$* constructs lacking the NTD are capable of forming RNPs of similar size, conceivably substituting NTD binding sites for nucleic acid by additional CTD subunits up-side-down orientation. (E) Mixed RNPs combining $N_{210\text{-}419}$* and full-length N protein different orientations.

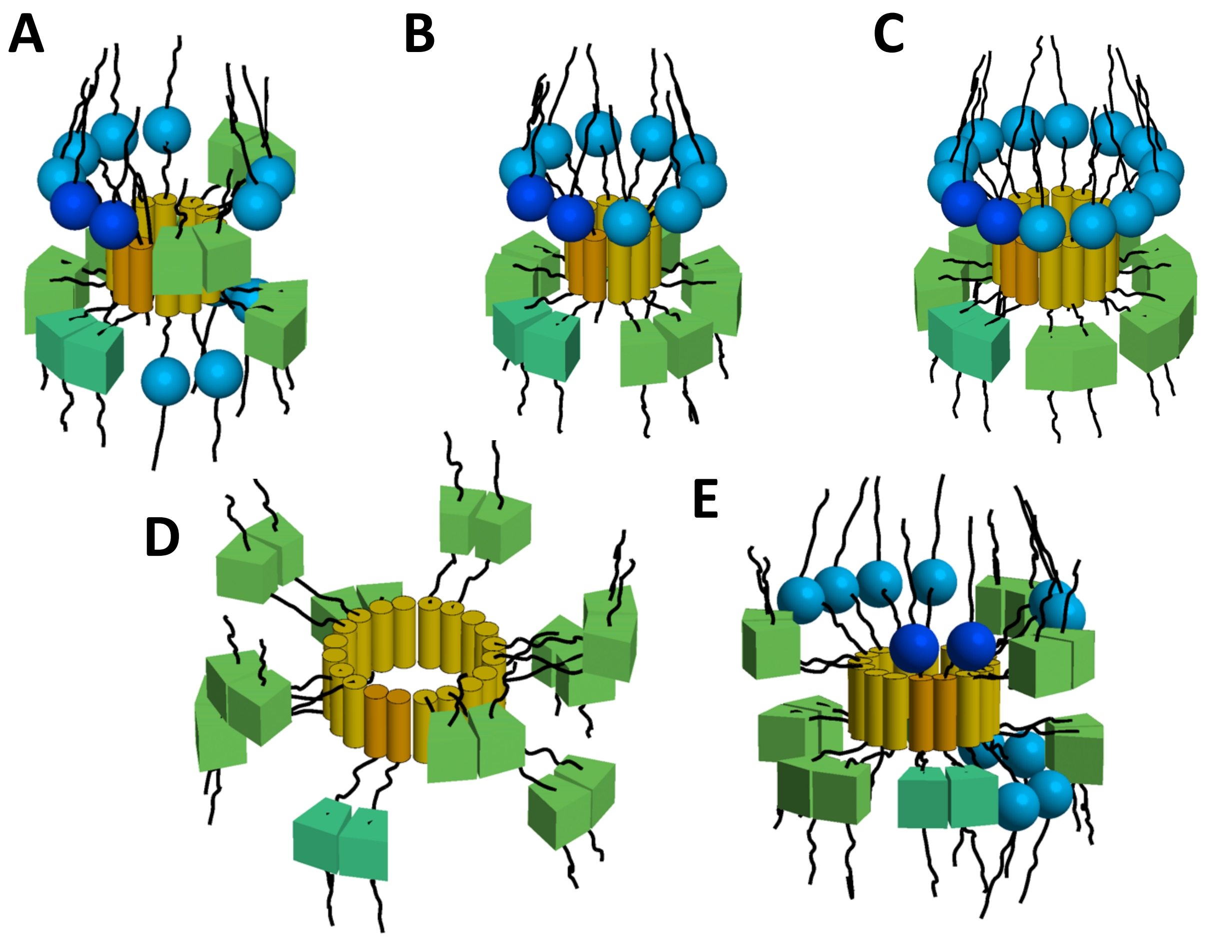

Supplement: Supplement 11 [file media-11.pdf]
