# Supplementary material for: Evolution of a fuzzy ribonucleoprotein complex in viral assembly
Source: bioRxiv. 2025 Nov 6:2025.04.26.650775. Originally published 2025 Apr 28. Preprint. [Version 3] doi: 10.1101/2025.04.26.650775 (PMC12190348; doi:10.1101/2025.04.26.650775)

Supplementary Figure S3: (A) Electron micrograph of negatively-stained N-arm peptide N_{1-43}:P13L after equilibration at 10 μM in 20 mM HEPES, 150 mM NaCl, pH 7.50. (B) As a control, electron micrograph of negatively-stained C-arm $N_{364-419}$ under the same conditions.

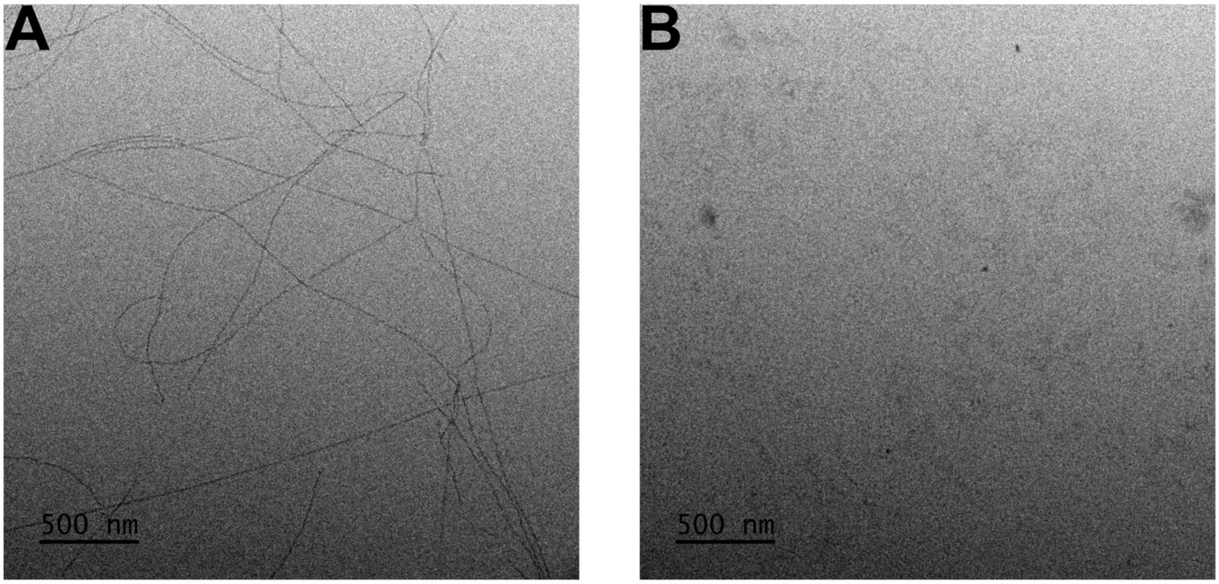

Supplement: Supplement 12 [file media-12.pdf]
