# Supplementary material for: Evolution of a fuzzy ribonucleoprotein complex in viral assembly
Source: bioRxiv. 2025 Aug 26:2025.04.26.650775. Originally published 2025 Apr 28. Preprint. [Version 2] doi: 10.1101/2025.04.26.650775 (PMC12190348; doi:10.1101/2025.04.26.650775)

**Supplemental Figure S10: Mutations of N:G214 and N:G215 across the phylogenetic tree of SARS-CoV-2**. Shown are all-time sequence samples in South America with clade labels and color-coded amino acid at position 214 and 215. The combination of 214C/G215 strain 21G (Lambda) is shown in blue, whereas the combination G214/215C of strain 21J (Delta) is shown in yellow. The phylogenetic tree was generated by Nextstrain (*2*).

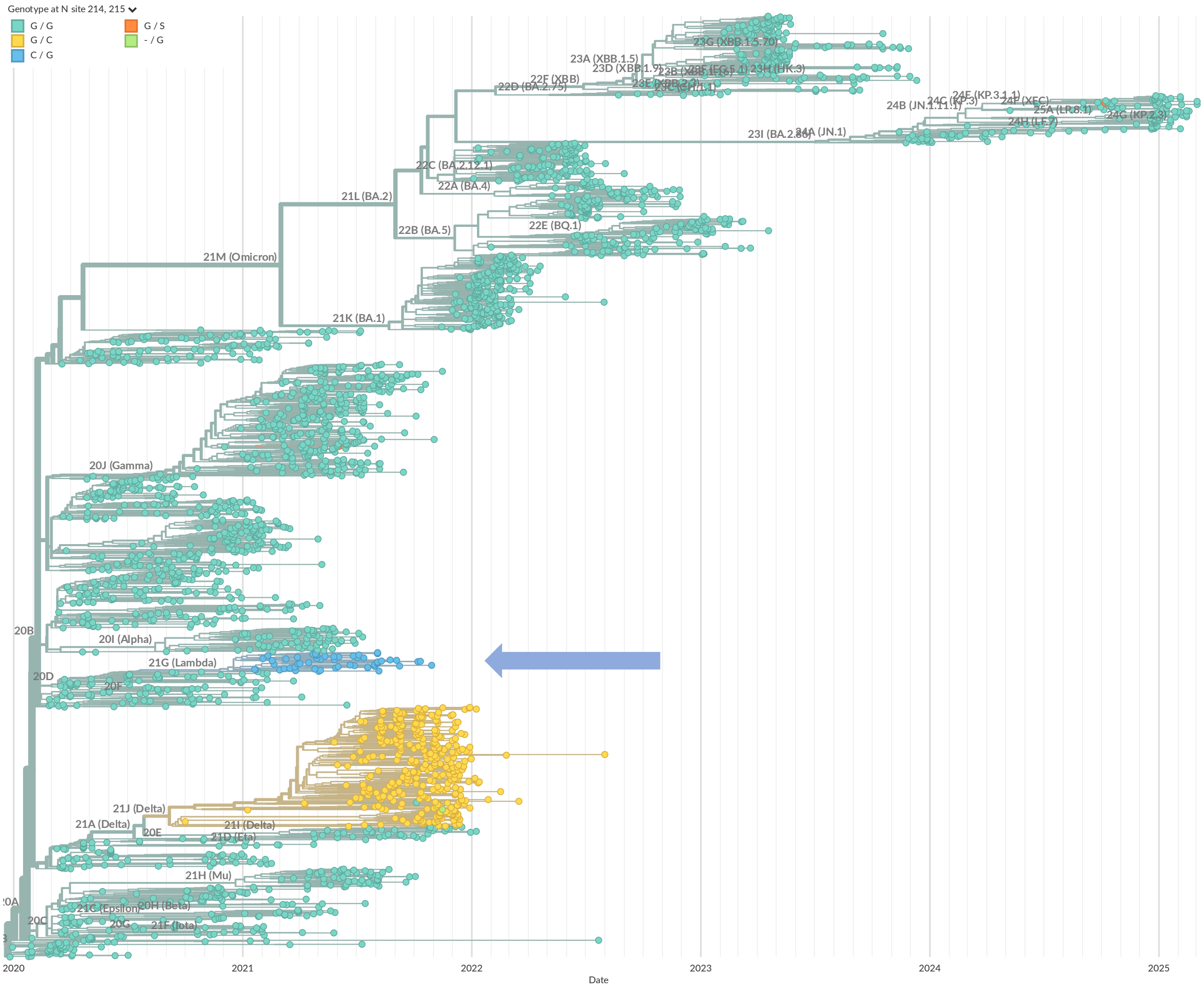

Supplement: Supplementary file 2 [file media-11.pdf]
