# Supplementary material for: Evolution of a fuzzy ribonucleoprotein complex in viral assembly
Source: bioRxiv. 2025 Aug 26:2025.04.26.650775. Originally published 2025 Apr 28. Preprint. [Version 2] doi: 10.1101/2025.04.26.650775 (PMC12190348; doi:10.1101/2025.04.26.650775)

Supplemental Figure S11: Possible heterogeneity of RNPs from alternate configurations of N dimer subunits. (A) Based on the cartoon of **Figure 1B**, an RNPs is sketched where two subunits have flipped LRS orientations, resulting in the swapped position of NTD and CTD subunits. (B) $N_{210\text{-}419}$* constructs lacking the NTD are capable of forming RNPs of similar size, conceivably substituting NTD binding sites for NA by additional CTD subunits up-side-down orientation. (C) Mixed RNPs combining $N_{210\text{-}419}$* and full-length N protein different orientations.

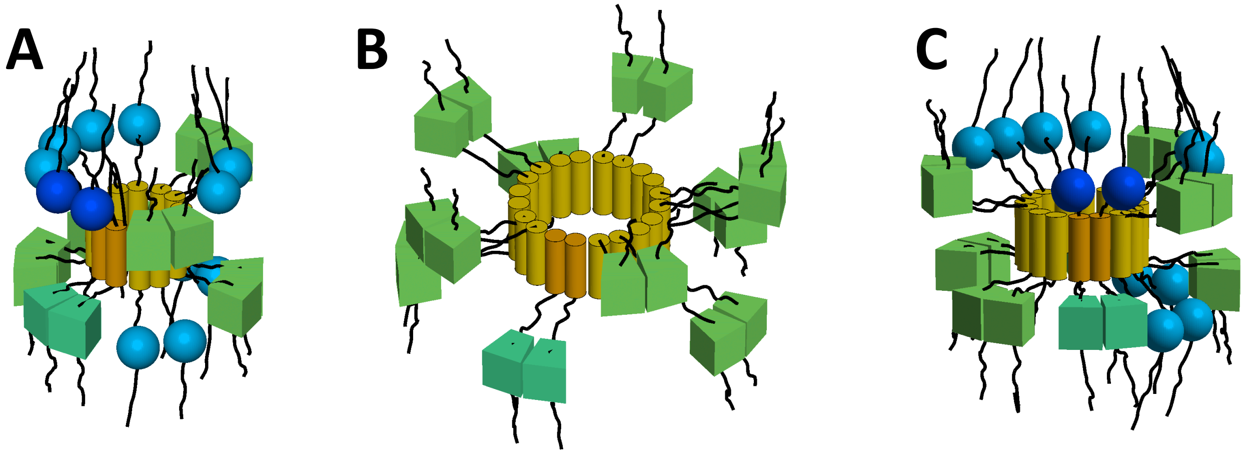

Supplement: Supplementary file 3 [file media-12.pdf]
