# Supplementary material for: Evolution of a fuzzy ribonucleoprotein complex in viral assembly
Source: bioRxiv. 2025 Aug 26:2025.04.26.650775. Originally published 2025 Apr 28. Preprint. [Version 2] doi: 10.1101/2025.04.26.650775 (PMC12190348; doi:10.1101/2025.04.26.650775)

Supplementary Figure S2: (A) Electron micrograph of negatively-stained N-arm peptide $N_{1\text{-}43}$:P13L after equilibration at 10 μM in 20 mM HEPES, 150 mM NaCl, pH 7.50. (B) As a control, electron micrograph of negatively-stained C-arm $N_{364\text{-}419}$ under the same conditions.

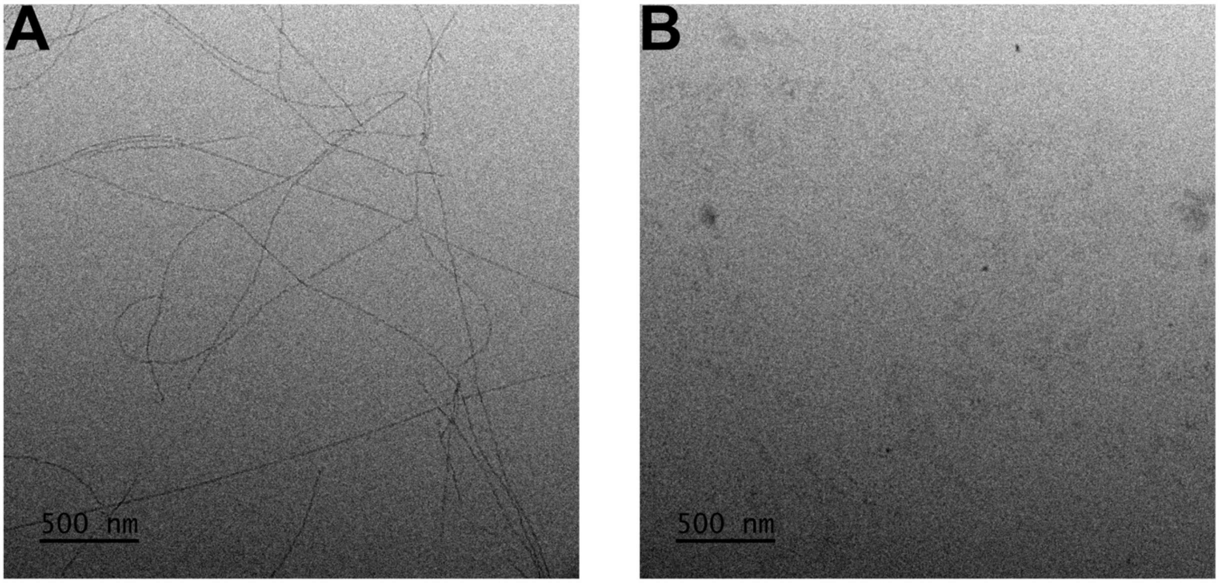

Supplement: Supplementary file 6 [file media-3.pdf]
