# Supplementary material for: Evolution of a fuzzy ribonucleoprotein complex in viral assembly
Source: bioRxiv. 2025 Aug 26:2025.04.26.650775. Originally published 2025 Apr 28. Preprint. [Version 2] doi: 10.1101/2025.04.26.650775 (PMC12190348; doi:10.1101/2025.04.26.650775)

Supplementary Figure S3: Comparison of WT and P13L structure predictions in ColabFold for a 12mer of the peptide $N_{10\text{-}20}$.

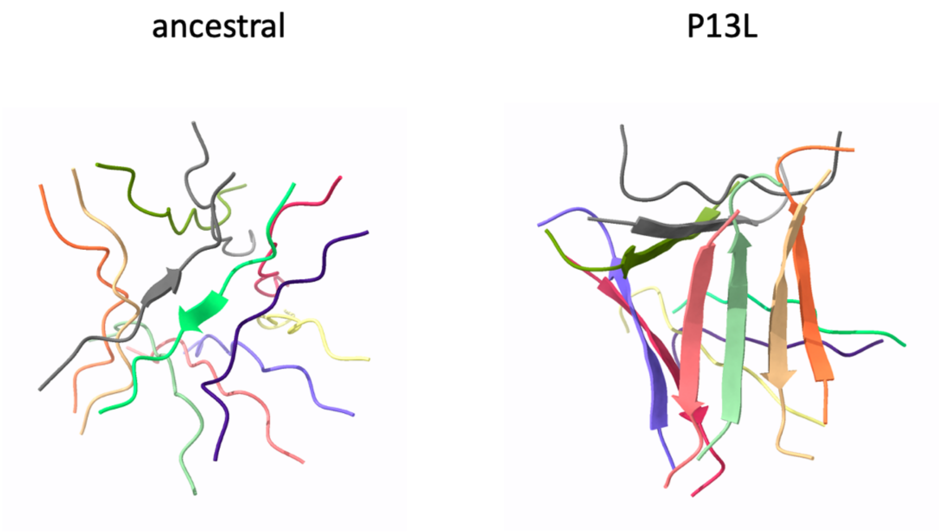

Supplement: Supplementary file 7 [file media-4.pdf]
