# Supplementary material for: Evolution of a fuzzy ribonucleoprotein complex in viral assembly
Source: bioRxiv. 2025 Aug 26:2025.04.26.650775. Originally published 2025 Apr 28. Preprint. [Version 2] doi: 10.1101/2025.04.26.650775 (PMC12190348; doi:10.1101/2025.04.26.650775)

Supplementary Figure S4: Size distribution of G214C cysteine mutant LRS peptides. Shown are peptides $N_{LRS,\ 210\text{-}246}$:G214C reduced (blue) vs unreduced (red). (A) Autocorrelation functions (circles) and best-fit size-distribution fits (solid lines). (B) Best-fit hydrodynamic radius distributions.

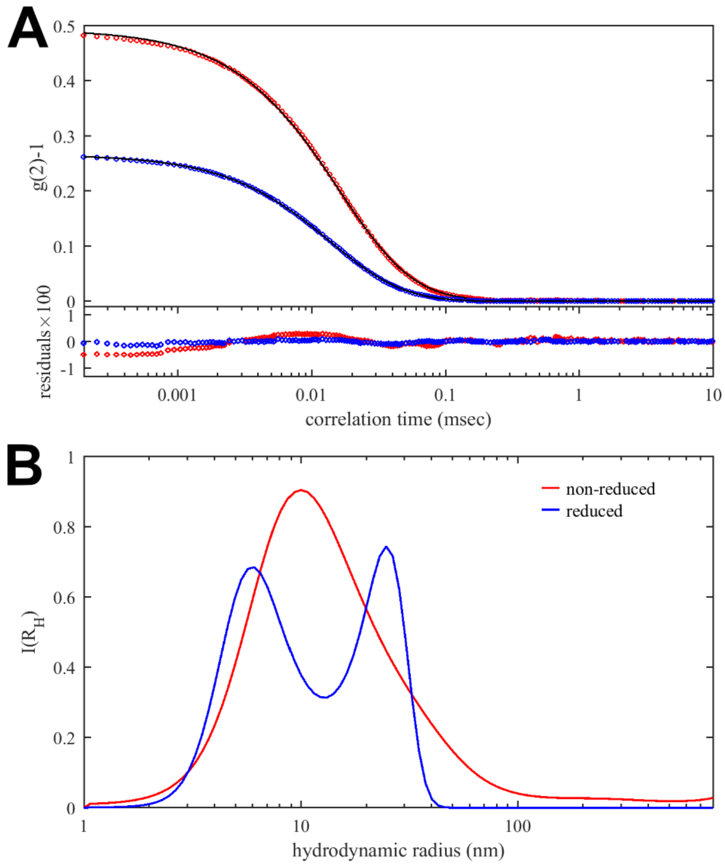

Supplement: Supplementary file 8 [file media-5.pdf]
