# Supplementary material for: Evolution of a fuzzy ribonucleoprotein complex in viral assembly
Source: bioRxiv. 2025 Aug 26:2025.04.26.650775. Originally published 2025 Apr 28. Preprint. [Version 2] doi: 10.1101/2025.04.26.650775 (PMC12190348; doi:10.1101/2025.04.26.650775)

Supplemental Figure S6: Non-reducing SDS-PAGE of reduced and oxidized N:G215C* and N$_{\lambda}$*. Lanes: 1) oxidized N$_{\lambda}$*; 2) reduced N$_{\lambda}$; 3) oxidized N:G215C*; 4) reduced N:G215C.

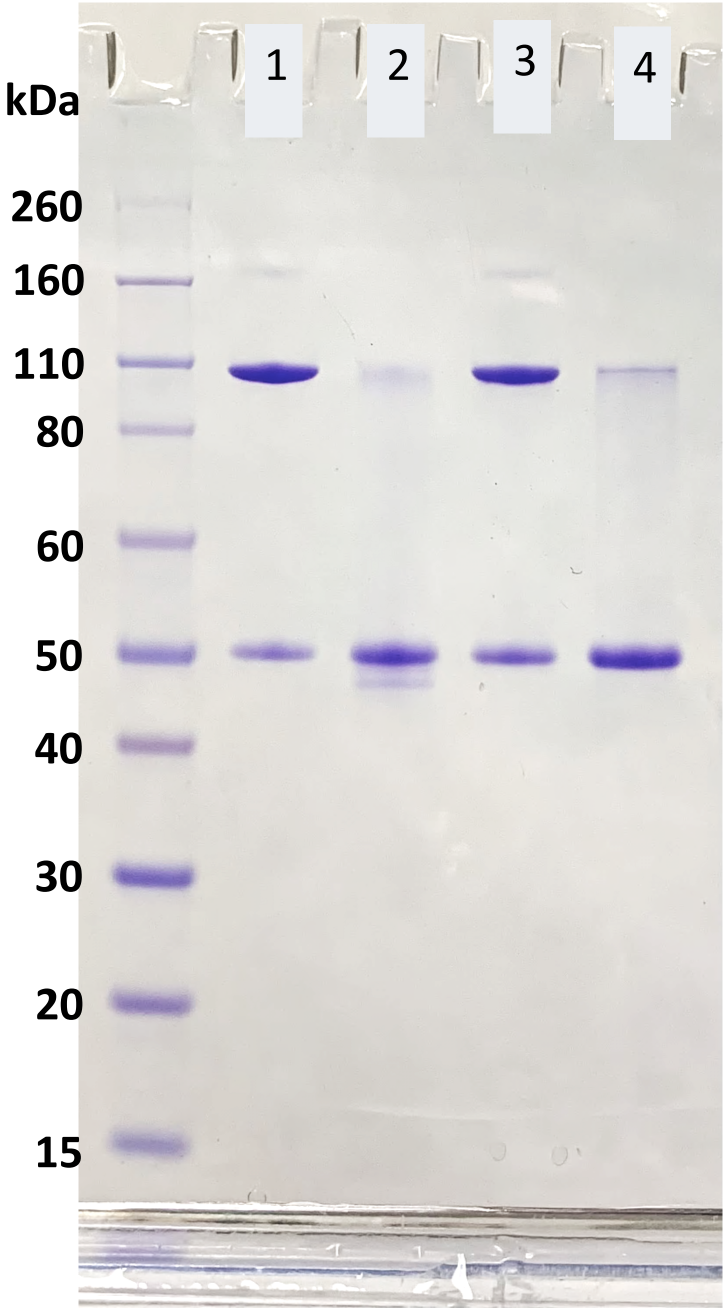

Supplement: Supplementary file 10 [file media-7.pdf]
