# Supplementary material for: Evolution of a fuzzy ribonucleoprotein complex in viral assembly
Source: bioRxiv. 2025 Aug 26:2025.04.26.650775. Originally published 2025 Apr 28. Preprint. [Version 2] doi: 10.1101/2025.04.26.650775 (PMC12190348; doi:10.1101/2025.04.26.650775)

Supplemental Figure S7: Measurement of the affinity of N:P13L,Δ31-33 for oligonucleotide T_{10} by SV-AUC titration. Sedimentation coefficient distributions are shown for 1.5 or 3.0 μM protein with T_{10} in different molar ratios. Integration of the overall weight-average *s*-value leads to the isotherm shown in the inset (circles), which is globally fitted with a binding model (lines) resulting in a K_D of 0.88 (0.70 – 1.1) μM. Experiments are in 20mM HEPES, 150mM NaCl, pH 7.5. In comparison, for ancestral N, the best-fit K_D-values and 95% confidence intervals are 1.1 [0.8–1.6] μM (*1*).

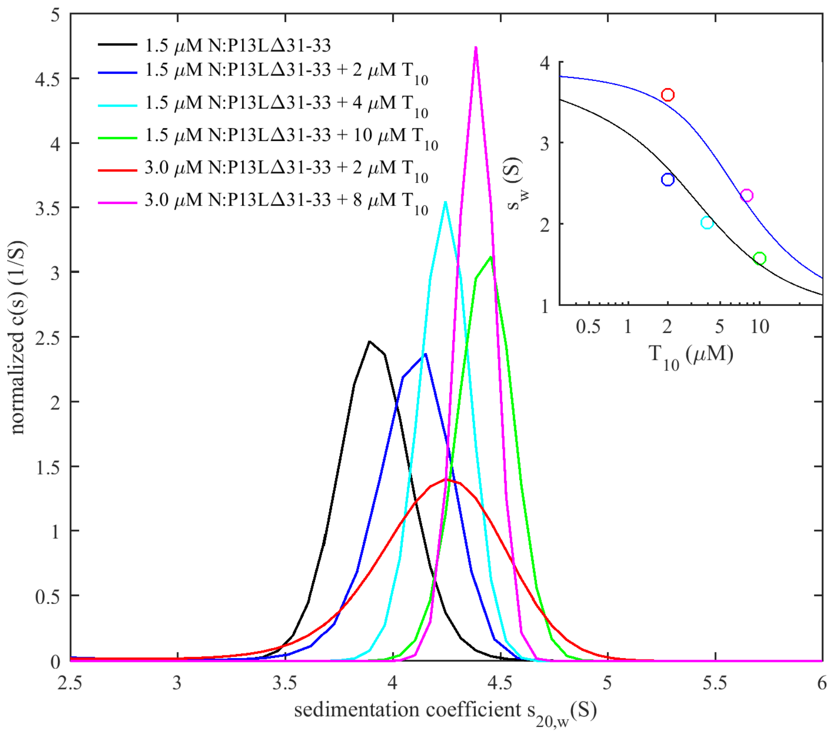

Supplement: Supplementary file 11 [file media-8.pdf]
