# Supplementary material for: Evolution of a fuzzy ribonucleoprotein complex in viral assembly
Source: bioRxiv. 2025 Aug 26:2025.04.26.650775. Originally published 2025 Apr 28. Preprint. [Version 2] doi: 10.1101/2025.04.26.650775 (PMC12190348; doi:10.1101/2025.04.26.650775)

Supplemental Figure S8: Mutations of N:P13 across the phylogenetic tree of SARS-CoV-2. Shown are all-time global sequence samples with clade labels and color-coded amino acid at position 13, with the ancestral P13 in green and P13L in yellow. The blue arrow points to the Lambda sequences. Additionally, a cluster of P13L mutations occurred in India in clade 19A. The phylogenetic tree was generated by Nextstrain (*2*).

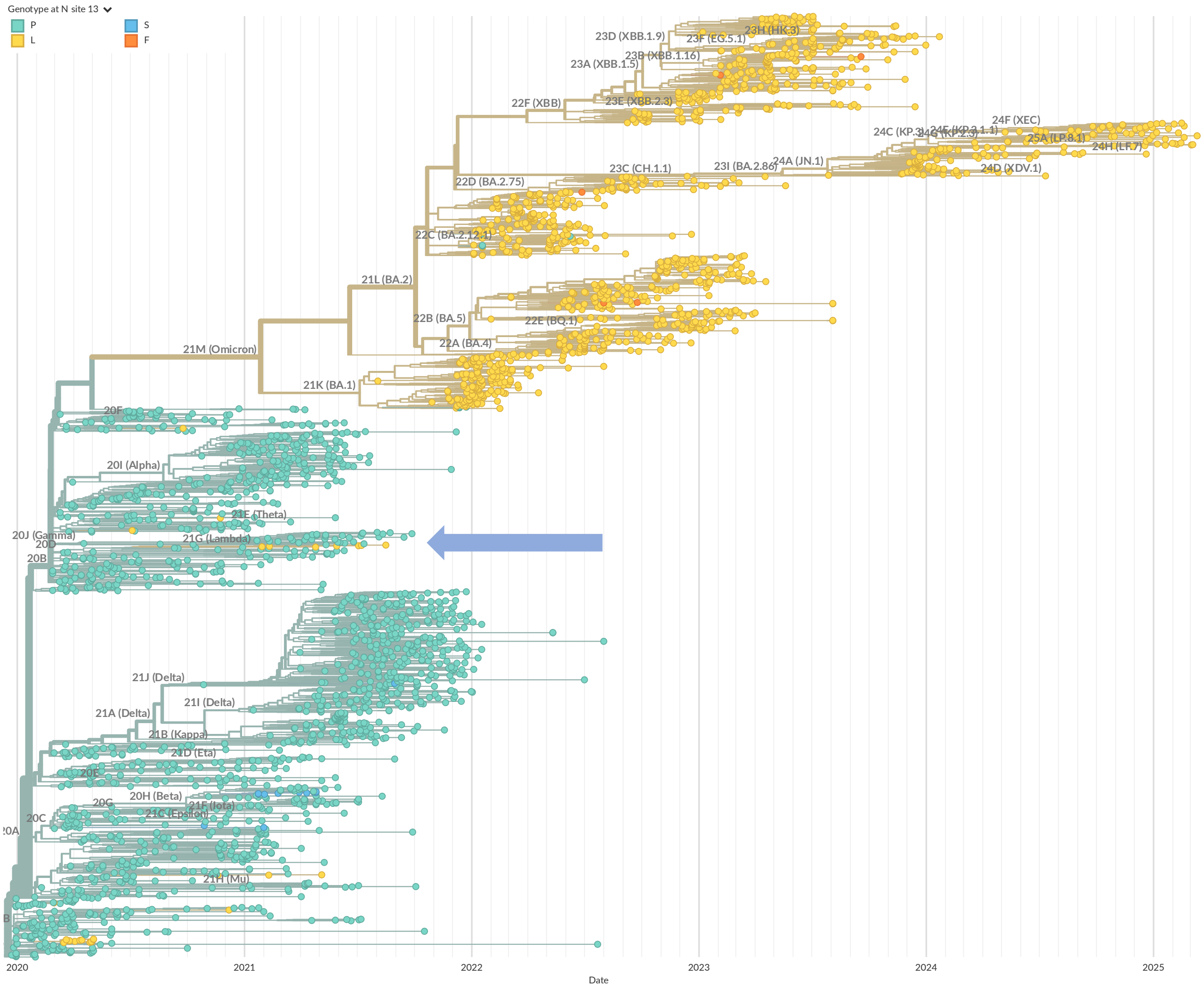

Supplement: Supplementary file 12 [file media-9.pdf]
